# Optimal production of *Phanerochaete chrysosporium* manganese peroxidases and *Trametes* sp. C30 laccase hybrid Lac131 in *Aspergillus niger* for lignin bioconversion

**DOI:** 10.1186/s13068-025-02690-x

**Published:** 2025-10-06

**Authors:** Ziyu Dai, Ana L. Robles, Sarah L. Lemmon, Guoliang Yuan, Dehong Hu, Jenny Onley, Jiayuan Jia, Kai Deng, Kshitiz Gupta, Trent R. Northen, Blake A. Simmons, Scott E. Baker, Jon K. Magnuson, Joonhoon Kim

**Affiliations:** 1 Joint BioEnergy Institute, Emeryville, CA 94608 USA; 2 Pacific Northwest National Laboratory, Richland, WA 99352 USA; 3 Lawrence Berkeley National Laboratory, Berkeley, CA 94720 USA; 4 Sandia National Laboratories, Livermore, CA 94550 USA; 5 Lawrence Livermore National Laboratory, Livermore, CA 94550 USA

**Keywords:** *Aspergillus niger*, *Trametes* sp. C30, Manganese peroxidase, Laccase hybrid Lac131, Transcriptional activator of proteases prtT, v-SNARE binding protein VSM, Bovine hemoglobin, Soy proteins, Skim milk

## Abstract

**Background:**

Incorporating the production of related ligninolytic enzymes into industrial filamentous fungus *Aspergillus niger* will enhance the bioconversion of lignocelluloses to various chemical products.

**Results:**

In this study, transgenic expression of *Phanerochaete chrysosporium* manganese peroxidases (*mnps*) and *Trametes* sp. C30 laccase hybrid Lac131 (*lac131*) were examined and optimized in *A. niger* 11414 *prtT*∆ strain. Five *mnps* (*mnp1*, *mnp2*, *mnp3*, *mnp4*, and *mnp5*) and *lac131* genes were expressed separately or in combination. The transgenic strain containing the entire *mnp2* genomic coding sequence (g*mnp2*) exhibited the highest mnP activity among the five *mnp* over-expression strains in the modified minimal medium (mMM) with addition of 5 g/L bovine hemoglobin (bHg). We examined the effects of hemin and bHg on mnP production in the g*mnp2* strain cultures and found that at least 1 g/L bHg was required, while hemin was not. Culture conditions for mnP production were further optimized for the g*mnp2* strain and the highest mnP activities were detected in the cultures grown at 25 °C and 200 rpm with an initial pH of 4.5. Effects of soy protein, skim milk, and bovine serum albumin on mnP production were investigated; 5 g/L of soy proteins or skim milk had comparable effects to 2.5 g/L bHg, while cultures with bovine serum albumin had diminished mnP activity. Disruption of both *prtT* and *vsm1* substantially augmented the mnP production and its activity reached 575 U/L. *Trametes* sp. C30 laccase hybrid *lac131* was strongly expressed in either *A. niger* g*mnp2* (1975 U/L) or 11414*prtT*∆ (3895 U/L) strain. Both mnP and laccase in the culture supernatants effectively decolorized selected phenolic compounds (dyes) and cleaved tagged model lignin dimers.

**Conclusion:**

The mnP was successfully produced in *A. niger* by optimizing the culture conditions and host strain. Co-expression of all four *mnp* genes in the same expression host by multiplex CRISPR will lead to the mnP production reaching levels comparable to *P. chrysosporium*, while only requiring 36 h at 25 °C. The Lac131 activity in transgenic *A. niger* strain is 4- to 7-times higher than that in previous studies. Co-production of mnP and laccase in *A. niger* will enhance the lignin bioconversion efficiency.

**Supplementary Information:**

The online version contains supplementary material available at 10.1186/s13068-025-02690-x.

## Background

Lignocellulose is the major component of global plant mass, which consists of cellulose, hemicellulose, and lignin. Lignin is a complex aromatic heteropolymer comprising three monolignol [ρ-hydroxyphenyl (H), guaiacyl (G), and syringyl (S)] units linked via a variety of ether and carbon–carbon linkages. An energy-dense heterogeneous polymer, lignin is closely intertwined with the cellulose and hemicellulose in plant cells walls and composes 15–40% of lignocellulosic biomass, making it the second most abundant biopolymer on earth after cellulose [1]. Breakdown of plant lignocellulose to glucose monomers is the basis for second-generation cellulosic bioethanol production, but the lignin polymer is highly resistant to depolymerization. Therefore, there is considerable interest in establishing methods to decompose and transform the lignin to value-added products.

Currently, methods for releasing monomeric sugars from lignocellulosic biomass include a DMR (deacetylation and mechanical refining) process along with enzymatic hydrolysis [2], a two-step process combining mechanochemical hydrolysis and diluted acid hydrolysis [3], and an aqueous ionic liquid at low severity process conditions [4]. These and other deconstruction methods effectively liberate high yields of monomeric sugars from the biomass for bioethanol production or other biorefineries and generate large amounts of lignin in insoluble and heterogeneous forms. In addition, large quantities of lignin are produced from the paper and pulping industry, which is simply burned for heat and power supply [5–8].

Lignin, like other lignocellulosic components, must be depolymerized before use in most industrial applications. Two types of approaches are currently used for lignin depolymerization: biological depolymerization and thermochemical depolymerization such as oxidation used for vanillin production [9]. Challenges of chemical methods include catalyst selectivity, relatively high temperature requirements, and repolymerization. In nature, various microorganisms (mainly fungi and bacteria) are capable of degrading lignin by utilizing lignin modification enzymes [10]. Previous studies on the microbial degradation of lignin have primarily focused on the breakdown by white-rot fungi that are able to mineralize lignin and release CO_2_ [11, 12]. White-rot fungi produce a range of extracellular ligninolytic enzymes, including manganese peroxidases (mnPs; E.C.1.11.1.13), lignin peroxidases (LiPs; E.C.1.11.1.14), versatile peroxidases (VPs: E.C.1.11.1.16), and laccases (Lacs: E.C.1.10.3.2). However, different white rot fungi produce different sets of ligninolytic enzymes; *Phanerochaete chrysosporium* mainly produces both manganese peroxidase and lignin peroxidase [13], while *Pleurotus eryngii* is known for production of versatile peroxidase [14] and *Trametes* sp. C30 for production of laccases [15].

Despite the continued study of fungal lignin degradation since the discovery of ligninolytic enzymes in the early 1980s [11, 16–18], there are a limited number of large-scale applications for biocatalytic process to depolymerize lignin, in part due to the practical challenges of the protein production via natural process or genetic engineering. Additionally, lignin repolymerization has been observed with prolonged incubation of lignin in certain fungal secretomes, which was attributed to intermediates of aldehydes and quinones and partially prevented by bacterial degradation of depolymerized lignin [19]. A combination of depolymerization and conversion of lignin to the selected value-added products within the same microorganism would be an effective way to reduce the lignin repolymerization, which has not yet been established. Therefore, a proper microorganism will need to be identified or created for this application.

The filamentous ascomycete fungus, *Aspergillus niger,* is an industrial production host for both organic acids and enzymes, making it an attractive candidate microbe to be evaluated for biological conversion of lignin to products. However, since *A. niger* does not produce the active ligninolytic enzymes (e.g., mnPs, LiPs, Lacs, and VPs), the first step is to define the optimal co-transgene expression of the ligninolytic enzymes in *A. niger*. Manganese peroxidase, one of the well-studied ligninolytic enzymes, has been characterized in both structure and functionality [20]. The *P. chrysosporium* manganese peroxidase (*pcmnp2*) was functionally previously expressed in *A. niger* with the supplement of bovine hemoglobin (bHg) or hemin in the cultures [21, 22]. Lignin peroxidase or laccase was also evaluated for transgene expression in *A. niger* [23, 24]. However, scaling up production of these ligninolytic enzymes in microbial cell factories, such as an industrial strain *A. niger* to meet various industrial demands has not been demonstrated, possibly due to the generation of highly reactive and nonspecific free radicals that may impede growth. Therefore, it is crucial to optimize both in vivo and in vitro production conditions for these industrial enzymes in *A. niger*.

In the present study, all five *P. chrysosporium mnp* (isoform: *mnp1*, *mnp2*, *mnp3*, *mnp4*, and *mnp5*) genes were examined for their functional expression in *A. niger* and it was demonstrated that four of five *mnp* genes can be functionally expressed. The effects of transcription factor *prtT* and the homolog of yeast v-SNARE-master1 (*vsm1*) on mnP production were also assessed in the mnP production transgenic strain pZD4175-8 by gene disruption, which synergistically resulted in substantial improvement of mnP production. Furthermore, a laccase hybrid (*lac131*) from *Trametes* sp. C30 was highly expressed in the transgenic strain of pZD4175-8 and *prtT*∆ and conditions were optimized for maximal expression. We demonstrate their functionality in decolorization of selected phenolic compounds (dyes) and cleavage of tagged model lignin dimers. These findings lay a foundation for a direct biological conversion of lignin to value-added chemicals.

## Materials and methods

### Strains and media

The *Escherichia coli* strain Top10 was used for routine plasmid DNA preparation. *A. niger* (ATCC 11414) from the American Type Culture Collection (Rockville, MD, USA), was grown on complete medium (CM) plates at 30 °C for culture maintenance and spore preparation. Deionized water (H_2_O) from Milli-Q Advantage A10 water purification system (MilliporeSigma, Burlington, MA, USA) was used throughout this work. Approximately 1x10^4^ to 1x10^5^ spores were inoculated on CM agar (petri dish) plates and incubated for 4 days at 30 °C. Spores were harvested by washing with 5–10 ml sterile H_2_O containing 0.4% Tween 80 (polyoxyethylene sorbitan monooleate) and pelleted by centrifugation at 2500 g for 5 min. The spores were re-suspended in sterile 0.4% Tween 80 H_2_O and enumerated with a hemocytometer. Aliquots of the resulting spore suspension (approximately 5x10^5^ to 1x10^6^ spores/ml) were used to inoculate liquid cultures. CM and minimal medium (MM) were prepared as previously described [25]. For enzyme production, the modified minimal medium (mMM) was prepared with regular *A. niger* minimal medium supplemented with 5 g/L casamino acid and 50 g/L maltose and adjusted the pH to pH 3.5, 4.5, 5.5 or 6.5. Different volumes of bHg sterilized by 0.2 μm filter or autoclave were added into the cultures to determine their effects on overall mnP or Lac131 production. Hemin chloride was dissolved in 0.1 M NaOH, neutralized to pH 7 with 1M HCl, adjusted to concentration of 5 mg/mL (7.7 mM) with sterilized distilled H_2_O, and sterilized by autoclave. Bovine serum albumin (BSA, 50 g/L) was sterilized by 0.2 μm filter filtration. Skim milk proteins (skimMPs; 50 g/L) and soy proteins (soyPs; 50 g/L) were sterilized by autoclaving at 121 °C for 15 min. Three different ways were used to prepare the soyPs. The first one was prepared as mentioned above. The second one was the autoclaved soyPs solution that was centrifuged at 20,000 g for 10 min to remove the insoluble materials. The third one was the unsterile soyPs solution that was centrifuged to discard the insoluble materials and sterilized with 0.2 μm sterile filter. All strains used in this study are shown in Table 1.

**Table 1:** Strains used in this study

Strain	Genotype	References
ATCC11414	Parent strain	Dai et al. [31]
pZD4100 (11414*prtT*∆)	*prtT*∆::*ble*	This work
BSC083 (comnp1)	*prtT*∆::*ble*, *gpdA*p:co*mnp1*:*gpdA*t, *hph*	This work
pZD4172 (ocmnp2)	*prtT*∆::*ble*, *gla1*p:co*mnp2*:*gpdA*t, *hph*	This work
pZD4173 (S*gla1-*comnp2-)	*prtT*∆::*ble*,*gla1*p:S*gla1*:co*mnp2*:*gpdA*t, *hph*	This work
pZD4174 (gmnP4)	*prtT*∆::*ble*, *gla1*p:g*mnp4*:*gpdA*t, *hph*	This work
pZD4175 (gmnP2)	*prtT*∆::*ble*, *gla1*p:g*mnp2*:*gpdA*t, *hph*	This work
pZD4175 (gmnP2, 11,414)	*gla1*p:g*mnp2*:*gpdA*t, *hph*	This work
pZD4176 (cmnP2/1st intron)	*prtT*∆::*ble*, *gla1*p:orig-c*mnp2*:*gpdA*t, *hph*	This work
pZD4180 (gmnP2-gfp)	*prtT*∆::*ble*,*gla1*p:g*mnp2-gfp*:*gpdA*t, *hph*	This work
pZD4181 (gmnP3)	*prtT*∆::*ble*, *gla1*p:g*mnp3*:*gpdA*t, *hph*	This work
pZD4198 (gmnP5)	*prtT*∆::*ble*, *gla1*p:g*mnp5*:*gpdA*t, *hph*	This work
pZD4206 (g*mnp2*/colac131)	*prtT*∆::*ble*, *gla1*p:g*mnp2*:*gpdA*t, *hph, ubi1*p:oc*lac131*:*gpdA*t, *nat1*	This work
pZD4207 (colac131)	*prtT*∆::*ble*, *gla1*p: oc*lac131*:*gpdA*t, *nat1*	This work
pZD4175vsm1	*prtT*∆::*ble*, *gla1*p:g*mnp2*:*gpdA*t, *hph, vsm1*∆	This work

### Preparation of transgene expression constructs for gene over-expression or gene disruption in *A. niger*

All transgene expressions or gene disruption constructs are shown in supplementary Fig. S1. The 1st transgene expression construct was prepared for *mnp1* (isoform H3) via de novo synthesis by GenScript Biotech Corp (Piscataway, NJ, USA), which contains a 1044 bp 5’-untranslated region (5’-UTR) of *A. niger* glucoamylase (*gla1*), 727 bp of *A. niger gpdA* (glyceraldehyde-3-phosphate dehydrogenase) promoter, the entire cDNA (1137 bp) of *P. chrysosporium mnp1* (co*mmp1*) gene with codon usage optimization for *A. niger*, 618 bp *Aspergillus nidulans trpC* transcriptional terminator, 1650 bp *hph* marker gene from pCSN44 [26], and 1000 bp 3’-UTR of *A. niger gla1* (BSC083). The gene disruption construct pZD4110 was assembled with the PCR fragments of the upstream and downstream regions of *prtT* gene (oligo pair: 2515prtTF1/2516prtTR1; 2519prtTF2/2520prtTR2, Table S1) and phleomycin *ble* marker gene (2517bleF/2518bleR). The *prtT* disruption was confirmed by PCR with oligo pairs of 2521scrF1/ble-293 and Ble-292/2522scrR1. The transgene expression constructs for pZD4172 to pZD4207 were prepared with the same DNA fragments of promoter (*gla1*p), *A. niger gpdA* transcriptional terminator (*gpdA*t), and hygromycin B phosphotransferase (*hph*) marker gene [27]. The pZD4172 contains the entire cDNA of *mnp2* (isoform H4, co*mnp2*) that was de novo synthesized with *A. niger* codon usage optimization. The PCR fragments for the *gla1*p, co*mnp2*, *gpdA*t, and bacterial hygromycin B resistance (*hph*) gene were isolated with oligo pairs of 2883glaF/2893glaR, 2894comnp2bF/2895comnp2bR, 2896gpdAtF/2886ampR, and 2887hphF/2888hphR, which were assembled into pBlueScript SK^(−)^ linearized with the *Hin*dIII and *Pst*I restriction enzymes by Gibson assembly [28]. The pZD4173 was identical to pZD4172, except the mnP2 signal peptide was replaced with the *A, niger* Gla1 one. The new oligo pairs for pZD4173 were 2883glaF/2897glaR, and 2898comnp2bF/2895comnp2bR.

The pZD4174, pZD4175, and pZD4180 were prepared with the entire *mnp4* (allele of *mnp2*) and *mnp2* genomic coding sequence with same *hph* marker gene (2887hphF/2888hphR). The pZD4174 & pZD4175 for the *mnp4* & *mnp2* were prepared with new oligo pairs of 2883glaF/2931glaR, 2932mnp2F/2933mnp2R, and 2934gpdAtF/2886ampR. The pZD4176 was prepared with the de novo synthesized DNA fragment of original *mnp2* cDNA with its first intron. The oligo pairs for pZD4176 were 2932mnp2F/2909mnp2R and 2910gpdAtF/2986gpdAtR. The pZD4180 contains *mnp2*-*gfp* fusion expression. The oligo pairs for pZD4180 were 2932mnp2F/2966mnp2F, 2967gfpF/2968gfpR, and 2969hphF/2888hphR. The pZD4181 for *mnp3* (isoform H5) with genomic coding sequence was prepared with oligo pairs of 2883glaF/2954glaR, 2955mnp3F/2956mnp3R, and 2957hphF/2886ampR. The pZD4198 for *mnp5* (isoform H9) with genomic coding sequence was prepared with oligo pairs of 2883glaF/2958glaR, 2959mnp5F/2960mnp5R, and 2961hphF/2886ampR.

The pZD4206 and pZD4207 were prepared for transgene expression of *Trametes* sp. C30 laccase hybrid *lac131* [functional hybrid of lac1 and lac3 [29]] gene. The *lac131* cDNA was de novo synthesized with *A. niger* codon usage optimization. The pZD4206 was prepared with the oligo pairs of 2680nat1F/3061nat1_ubi1pR, 3062lac131F/3063lac131R, and 3064actrpctF/2675actrpctR. The pZD4207 was prepared with the oligo pairs of 3065gla1F/3066lac131R, 3067actrpctF/3068actrpctR, and 3069nat1F/3070nat1R. A plasmid-based CRISPR–Cas9 cassette with two guide RNA (gRNAs) was prepared and named pGY35.

### Chemical-mediated protoplast transformation of *A. niger*

The protoplast preparation and chemical-mediated transformation followed the method described previously for *A. niger* [30]. The transgene expression cassettes from selected plasmid DNAs shown in supplementary Fig. S1 were briefly linearized by the restriction enzymes *Bam*HI (BSC083), *Pvu*II (pZD4174, 4175, 4176, 4180, 4181, 4206, 4207), *Sac*I (pZD4110), *Kpn*I/*Xba*I (pZD4198), or *Xho*I/*Xba*I (pZD4172 & 4173). Ten microliters of the linearized plasmid DNA (about 2.0 μg) were used for *A. niger* protoplast transformation for the transgene over-expression cassettes. For the gene deletion construct of *prtT*, approximately 1 μg of plasmid DNA linearized by restriction enzyme *Sac*I was used for protoplast transformation in *A. niger*. The *vsm1* gene was disrupted via the CRISPR–Cas9 method, in which the 2 μg of intact supercoiled pGY35 plasmid was used for protoplast transformation. Approximately 15 transformed clones were typically picked randomly for evaluation of the mnP or Lac131 production based on enzyme activity assays.

### Spore square PCR and total genomic DNA isolation

Two methods were used to isolate the total genomic DNA for transgene expression or gene disruption confirmation. The first one was the cetyltrimethylammonium bromide (CTAB) extraction method. 50 to 100 mg of lyophilized biomass and two 3.5 mm diameter glass beads were transferred into a 2 mL polypropylene micro-vial, where biomass was pulverized into fine power with a Mini-Beadbeater-8 (Bio Spec Products Inc., Bartlesville, OK, USA) for 50 s. The disrupted cells in microcentrifuge tubes were re-suspended with 800–900 μl of CTAB solution and incubated at 60 °C for 30–45 min., occasionally being inverted. The genomic DNA in the supernatant of the cell extracts was extracted with 300 μl of phenol/chloroform solution and precipitated with 800 ml of 2-propanol. Further steps for the genomic DNA purification followed the procedures described previously [31]. Recently, a rapid and robust square PCR method was established in our lab [32], which was applied to confirm the *vsm1* (v-SNARE binding protein, *vsm1*∆) gene disruption in *A. niger* by CRISPR–Cas9 system.

### Culture methods

125 ml glass Erlenmeyer flasks (Pyrex) were prepared by filling with 5% Contrad 70 (Decon Labs, Inc. King of Prussia, PA, USA) and soaked overnight to remove any potential residues on the inside surface of flasks prior to general dishwashing. Silicon sponge closures were used for all flask cultures. The biomass of transgenic clones and parent strain for genomic DNA isolation were prepared from 2 mL stationary CM cultures with proper antibiotics and grown in 13 × 100 mm glass culture-tubes for 24–36 h at 30 °C. The biomass formed on the surface of the liquid culture medium was collected, frozen immediately in liquid nitrogen and dried in the VirTis benchtop manifold freeze dryer (SP Scientific, Gardiner, NY, USA).

For mnP or Lac131 production, 30 ml mMM were aliquoted into the 125-mL culture flask and inoculated with 3 × 10^7^ *A. niger* spores. Proper amounts of bHg, soyPs, skimMPs, BSA (bovine serum albumin), or hemin chloride were added into the *A. niger* mMM cultures. The cultures were grown in the incubator shaker set at 20, 25, 30, or 35 °C and the shaker speed at 150, 200, or 300 rpm.

### Enzyme assays

All culture supernatants used for enzyme assays were filtered through 1 layer of Miracloth (MilliporeSigma, Burlington, MA, USA). The mnP activity measurement followed the method described previously [33]. The mnP assay was carried out in 96-well flat bottom microplates with the SpectraMax M5 microplate reader (Molecular Devices, CA, USA). 30 μl of samples with a specific dilution and 205 μl of assay mixture (50 mM sodium succinate, pH 4.5, 50 mM L-lactate, 2 mM MnSO_4_, 3 mg/mL gelatin, and 0.14 mM ABTS [diammonium 2,2’-azinobis-3-ethylbenzthiazoline-6-sulfonate]) were added to each well, shaken for 5 s to mix, and incubated at 35 °C for 2 min. The ABTS oxidation was initiated by the addition of 15 μl 50 mM H_2_O_2_ and monitored at 410 nm for 3 min. The molar extinction coefficient (ε_410_) of 36,000 M^−1^ cm^−1^ was used to determine the enzyme activity.

The laccase hybrid (Lac131) activity measurement was also carried out in 96-well microplates with the method described previously [34], but modified for this study. 180 μl of assay mixture (100 mM sodium acetate pH5.0 and 2.0 mM ABTS) was aliquoted into the microplate wells and incubated at 35 °C for 2 min. The reaction was initiated by the addition of 20 μL of culture supernatant.

### Aromatic dye decolorization

In this study the synthetic dyes bromophenol blue (590 nm), brilliant blue (628 nm), crystal violet (592 nm), methyl red (435 nm), methyl orange (465 nm) and Remazol brilliant blue R (594 nm) were used to determine the efficiency of dye decolorization by mnP or laccase (Lac131). The stock solution of selected dyes (5 g/L) was prepared first. The working solution was prepared with 50 mM sodium acetate (pH5.0) and 30 μg/mL of selected dyes except crystal violet (15 μg/mL). Twenty-five microliters of culture supernatants were mixed with 975 μL of the working solution in 1.5 mL polystyrene cuvettes and incubated at 30 °C for 25 or 50 h. The dye decolorization was determined by its specific wavelength at different intervals. The decolorization rate (%) was calculated as:

1 $${\text{Decolorization rate}} = \left( {{\text{A}}_{{\text{i}}} - {\text{A}}_{{\text{t}}} } \right)/{\text{A}}_{{\text{i}}} *{1}00.$$

Here, A_i_ is the initial absorbance of the reaction at time *t* = 0, while A_t_ denotes the absorbance of reaction mixture at a given time t.

### NIMS activity and mass spectrometry imaging (MSI)

Nanostructure-initiator mass spectrometry (NIMS) assays were performed as described previously [35]. The *A. niger* culture supernatants filtrated with one-layer Miracloth (MilliporeSigma, Burlington, MA, USA) were additionally centrifuged for five minutes at 10,000 × g, and the resulting supernatants were diluted to a final concentration of 1–200 U/L enzyme in each reaction. Enzymes were diluted to a concentration at which dimer cleavage and product formation could be tracked over time. Reactions contained 0.5 mM NIMS substrate (model lignin dimers) and 50 mM buffer. Reactions with laccase contained sodium acetate buffer (pH 4.8). Reactions with mnP contained succinic acid buffer (pH 4.5), 5 mM lactate, 0.5 mM MnSO_4_, and 80 nM H_2_O_2_. Enzyme reactions were prepared in 96-well PCR plates, sealed with foil seals, and incubated at 30 °C (laccases) or 35 °C (mnPs). Samples were mixed 1:1 with 0.2% trifluoroacetic acid then transferred to the NIMS chip with a robotic liquid handler as previously described [35].

In brief, MSI was done using Bruker ultrafleXtreme MALDI-TOF mass spectrometer over a mass range of 200–3500 Da. The imaging method included a 175-μm gap between adjacent laser shot locations and each location was shot 400 times. Spectra were recorded in positive reflector mode. The instrument was calibrated using Anaspec Peptide Calibration mixture 1 (catalog #AS-60882; Anaspec, Fremont, CA). Spectra were exported to OpenMSI [36] and all pixels containing peaks with a relative signal intensity of approximately ten times above background noise were analyzed. Peaks were binned into “substrate” (mass equivalent to substrate + 1) or “product” (intermediate and product peaks identified previously [35]. Relative proportions of the bins were calculated as the ratio of the sum total intensity of peaks in the given bin and the sum total intensity of peaks in all the bins. Data were handled with pandas Version 2.2.2 python package.

### Statistical analysis

The experimental data were presented as the mean ± standard deviation (SD). Statistical analyses were performed using the GraphPad Prism 10.0 software (GraphPad Software, Inc., San Diego, CA, USA). One-way analysis of variance (ANOVA) analysis was used to evaluate differences in mean values among three groups, followed by Tukey’s post hoc tests of multiple comparisons when ANOVA were significant. Statistical significance level was set at *p* < 0.05.

## Results and discussion

*A. niger* is well known as a versatile cell factory for organic acid and protein production. Integration of enzymatic lignin depolymerization into *A. niger* is a promising strategy for bioconversion of lignin to fuels or chemicals. It has been demonstrated previously that *P. chrysosporium mnp2* may be expressed in *A. niger*, requiring supplements of bHg or heme chloride [21]. The *mnp2* was also functionally expressed in *Pichia pastoris* and corn seeds without heme-related supplements [37, 38]. In this study, the transgene expression of the entire five isoforms of *P. chrysosporium mnp* genes were examined and further optimized. Additionally, the potential dual expression of *Trametes* sp. C30 laccase hybrid *lac131* and *mnp* together in the citric acid production *A. niger* strain was evaluated. Their potential capabilities in lignin deconstruction were assessed by decolorization of selected aromatic dyes and cleavage of tagged model lignin dimers.

### Transgene expression vectors prepared for investigation of mnP and laccase hybrid production in *A. niger*

Five different *mnp* (*mnp1* to *mnp5*) genes were identified previously [39]. One study enhanced the *mnp1* (isoform-1) homologous expression in *P. chrysosporium* [40]. In addition, *mnp2* has been evaluated for its functional expression in several organisms [21, 37, 38, 41]. In this study, we assess the five known isoforms of *P. chrysosporium mnp* genes for their ability to be expressed in *A. niger*. A series of transgene expression vectors was prepared (Fig. S1). The transgene expression of *P. chrysosporium mnp1* (isoform-1), *mnp2* (isoform-2), *mnp3* (isoform-3), *mnp4* (isoform-4, high homolog to *mnp2*), *mnp5* (isoform-5), and *lac131* of *Trametes* sp. C30 laccase hybrid were all under the control of *A. niger gla1* promoter (*gla1*p) and *gpdA* transcriptional terminator (*gpdA*t) except the *gpdA* promoter/trpC terminator for BSC083 and the *ubi1* promoter for pZD4206.

### Assessment of *P. chrysosporium* manganese peroxidase (*mnp*) transgene expression in *A. niger* 11414*prtT*∆ strain

Initially, the transgene expression cassette for the *mnp1* gene was transferred into *A. niger* and the transgene expression was determined via enzymatic assay using ABTS as a substrate. No mnP activity was detected in the culture supernatants collected from samples of 15 different transgenic strains under various culture conditions and durations. Many potential factors may impact expression in *A. niger*, such as degradation in the culture by secreted proteases. The *prtT* gene is known to be involved in the regulation of extracellular protease expression [42]. To improve the mnP over-expression in *A. niger*, the *prtT* was first disrupted and confirmed by PCR (Fig. S2). The five transgene expressions were subsequently examined in the *A. niger* 11414*prtT*∆ strain. The *mnp2* transgene expression with or without *gfp* fusion in *A. niger* was first visualized microscopically. Indeed, the GFP in the mycelia was detected in the transgenic *A. niger* strain with the *mnp2*-*gfp* fusion expression (Fig. S3), suggesting that the *mnp2*-*gfp* was properly expressed in *A. niger*. Therefore, the transgene expression of *mnp2* along other selected *mnp* genes was further evaluated in *A. niger* 11414*prtT*∆ strain in depth.

### The effects of different culture conditions on mnP production in *A. niger*

**Fig. 1:**
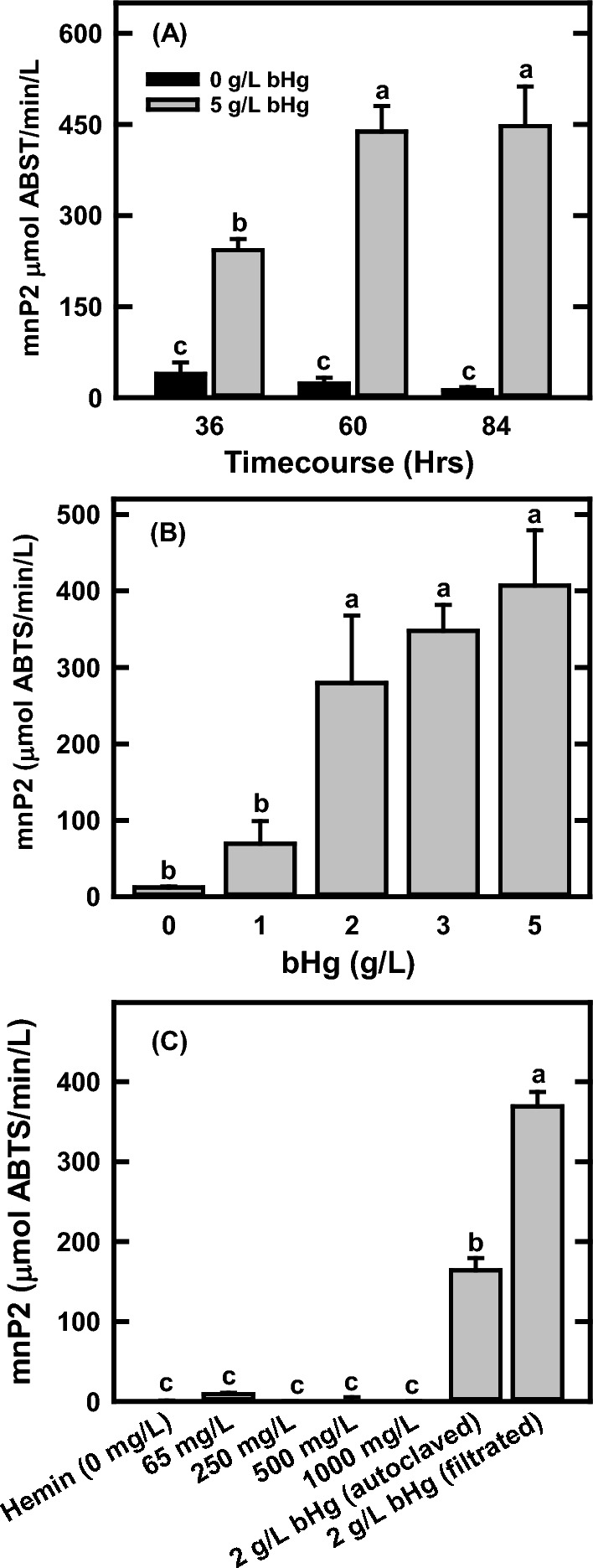
The effects of bovine hemoglobin and hemin on manganese peroxidase (mnP) production in the *mnp2* transgenic strain pZD4175-8 grown in the modified minimal medium (mMM). **A** Time-course of mnp2 production in the mMM with or without 5 g/L of bovine hemoglobin at 30 °C and 200 rpm. **B** The effects of different concentrations of bovine hemoglobin on mnp2 production in the mMM at 30 °C and 200 rpm after 2 days growth. **C** The effects of different concentrations of hemin on mnp2 production in the mMM at 30 °C and 200 rpm after 2 days growth as compared to the cultures with g/L of bovine hemoglobin prepared by direct autoclave or 0.2 μm filtration. All data represent mean ± SD (*n* = 3 biological replicates). Different lowercase letters represent statistically significant differences (*p* < 0.05) using ANOVA and Tukey’s post hoc tests

Bovine hemoglobin (bHg) and Hemin: A previous study demonstrated that both hemin and bHg have positive effects on mnP production in transgenic *A. niger* [21]. In this study, one of the transgenic strains (pZD4175-8, mnP2) with the highest mnP activity was selected to define optimal conditions for mnP production in shake flask cultures (Fig. 1). When the transgenic *A. niger* strain was grown with the modified minimum medium (mMM) without additional bHg, the mnP activity was approximately 39 μmol ABTS/min/L [Hereafter, one unit (U) is defined as 1 μmol ABTS/min] after 36 h of growth at 30 °C and 200 rpm. This decreased to 12 U/L after 84 h. In contrast, the mnP activity in the culture with 5 g/L of bHg was about 243 U/L at 36 h and reached 438 U/L after 60 h of growth (Fig. 1A). No obvious improvement was observed after growth for an additional 20 h. The effects of different bHg concentrations on mnP production were examined as well (Fig. 1B). The mnP activities were from 12, 69, 280, 348, to 407 U/L in the cultures with 0, 1, 2, 3, and 5 g/L bHg after 2 days growth, respectively. The effects of hemin on mnP production were also assessed. About 0, 70, 250, 500, and 1000 mg/L hemin chloride were added into the mMM culture medium and grown for 2 days. The mnP activities were barely detected (ranging from 0.2 to 9 U/L) in those cultures (Fig. 1C), in contrast with previous observations [21], suggesting the extracellular heme is not required for mnP functional expression in *A. niger*. The heme is an iron-containing molecule that serves as a prosthetic group in mnP and can be produced endogenously. Although recent study has demonstrated that heme transporter homologs were identified in *Aspergillus fumigatus* [43], what we observed may be due to the heme solubility in the culture broth and its import efficiency in our *A. niger* strains. The effects of bHg preparations on the mnP activity in the cultures were also investigated. The results in Fig. 1C show that the mnP activity in the cultures with autoclaved bHg was less than 50% of that with 0.2-micron filtration.

**Fig. 2:**
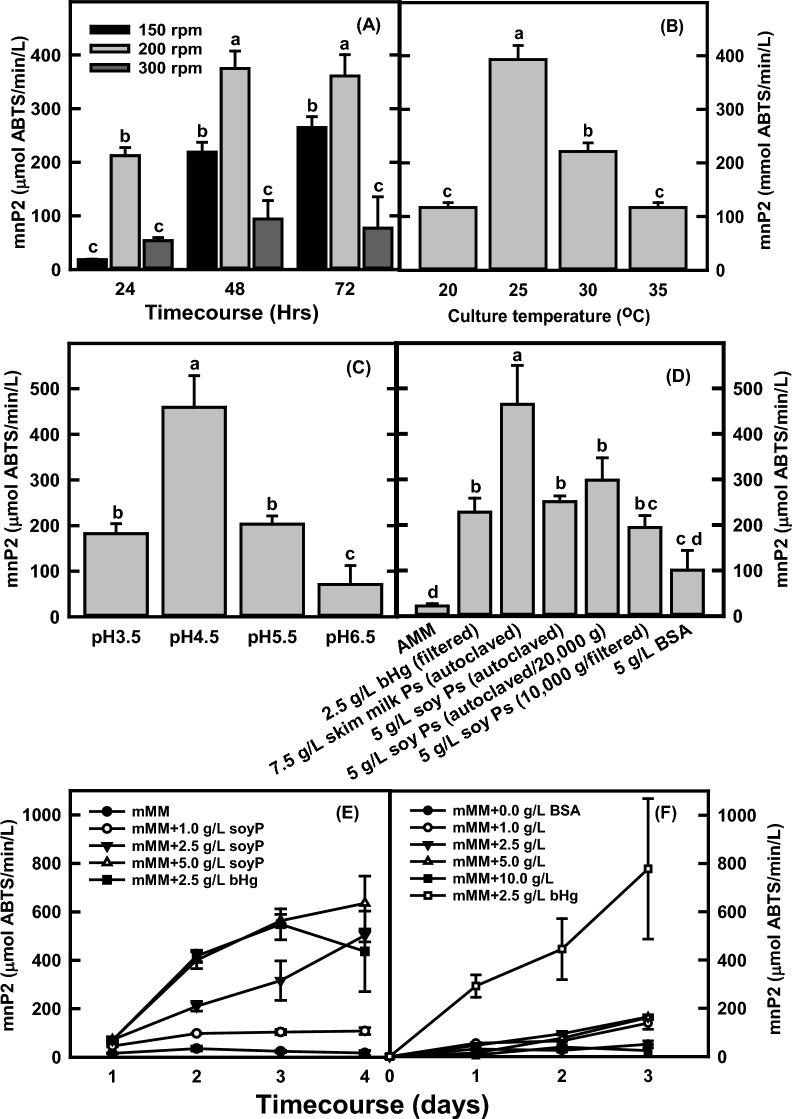
The effects of shaker speed, culture temperature, initial medium pH, skim milk (skim Ps), soy proteins (soy Ps), and bovine serum albumin (BSA) on manganese peroxidase (mnP) production in *mnp2* transgenic strain pZD4175-8 grown in the modified minimal medium (mMM). **A** Time-course of mnP production in the 5 g/L bovine hemoglobin mMM at 30 °C with the shaker speeds of 150, 200, or 300 rpm; **B** the culture temperature effects on mnP production in the 5 g/L bovine hemoglobin mMM at the shaker speed of 200 rpm after 2 days growth; **C** mnP production by the *mnp2* transgenic strain pZD4175-8 in the various initial pH in the 5 g/L bovine hemoglobin mMM after 2 days growth; **D** the effects of skim milk, soy proteins (autoclave versus filtration), and BSA on manganese peroxidase (mnP) production in *mnp2* transgenic strain pZD4175-8 grown in the modified minimal medium (mMM) at 30 °C and 200 rpm after 2 days growth; **E** time-course of mnp2 production in the mMM with 1, 2.5, or 5 g/L of soy proteins (soyP) and 2.5 g/L bovine hemoglobin at 30 °C and 200 rpm; **F** time-course of mnp2 production in the mMM with 1, 2.5, 5, or 10 g/L of BSA and 2.5 g/L of bovine hemoglobin at 30 °C and 200 rpm. All data represent mean ± SD (*n* = 3 biological replicates). Different lowercase letters represent statistically significant differences (*p* < 0.05) using ANOVA and Tukey’s post hoc tests (**A**, **B**, **C**, **D**)

Shaker speed, temperature, and pH: Shaker speed, temperature, and pH effects on mnP production with 5 g/L filtered bHg in mMM were investigated using the pZD4175-8 transgenic strain. Figure 2A shows that the mnP activities in the culture were 18, 219, and 265 U/L at 150 rpm, 212, 375, and 361 U/L at 200rmp, and 54, 94, and 77 U/L at 300 rpm after 24, 48, and 72-h growth, respectively. The results indicate that the optimal speed of shaker incubator for the mnP production is 200 rpm. The effects of culture temperatures on mnP production were evaluated in the shaker flask culture after 2 days growth (Fig. 2B). The mnP activities were 117, 393, 221, and 117 U/L in the cultures grown at 20, 25, 30, and 35 °C, respectively. The optimal temperature for mnP production was therefore 25 °C. The effects of initial pH in the culture medium on mnP production were quantified (Fig. 2C). The mnP activities in the cultures with initial pH 3.5, 4.5, 5.5, or 6.5 were 182, 459, 230, or 75 U/L, respectively. The optimal initial pH in the culture medium with filtered bHg was 4.5.

The impact of soy proteins (soyPs), skim milk (skimPs), and BSA on mnP production: Since the hemin did not improve mnP production in the cultures and the cost for both bHg and filtered preparation is high, alternative protein sources for mnP production were evaluated. The soyPs, skimPs, and BSA were selected to examine their effects on mnP production in mMM culture medium. The results in Fig. 2D show that additions of soyPs, skimPs or BSA into the cultures significantly improved mnP production in the cultures of *mnp2* transgenic strain. The mMM cultures were supplemented with 2.5 g/L of filtered bHg, 7.5 g/L autoclaved skimPs, 5 g/L soyPs from 50 g/L concentrate suspensions prepared with autoclave only, supernatants from centrifugation after autoclave or centrifugation and filter sterilization, or 5 g/L BSA. The mnP activities in culture supernatants reached 228, 464, 251, 298, 195, and 101 U/L, respectively. Both skimPs and soyPs had positive effects on mnP production compared to bHg. In contrast, the 5 g/L BSA was less effective than that of 2.5 g/L bHg, 7.5 g/L skimPs or 5 g/L soyPs.

The effects of soyPs or BSA on mnP production at different concentrations were also determined over 3 or 4 days growth. The results in Fig. 2E show that adding 1, 2.5 or 5 g/L of soyPs into the cultures significantly increased mnP activities ranging from 17, 108, 503, to 636 U/L after 4 days of growth. The mnP activity curve in the culture with 5 g/L soyPs was similar to the culture with 2.5 g/L filtered bHg proteins. In contrast, the mnP activities in cultures with 0, 1, 2.5, 5.0, or 10 g/L BSA were 51, 138, 165, 162, and 25 U/L, respectively (Fig. 2F). The addition of 1 g/L BSA into the cultures increased mnP activity by approximately 2.7-fold. Still, no significant improvement was observed with additional amounts of BSA. This suggests that the bHg from bovine erythrocytes or soyPs from the defatted soy meal may provide more abundant protease cleavage sites than the BSA to reduce the mnP degradation in *A. niger*.

The molecular mechanisms are unknown for the mnP production improvement by additions of bHg, skimPs, soyPs, or BSA into the *A. niger* cultures. It may be due to reduction of mnP degradation by extracellular proteases or cellular damage by mnP in the cultures. Therefore, inexpensive sources of proteins such as soyPs (~ 25 times cheaper than bHg) are viable options to be used for mnP production in *A. niger*.

### The effects of codon usage, intron, signal peptide or intact genomic coding region on mnP production in *A. niger*

The *mnp2* from *P. chrysosporium* was examined for transgene expression in several organisms such as *E. coli*, *A. niger*, *A. oryzae*, *P. pastoris,* and corn seed [21, 37, 38, 44]. To maximize the mnP production in *A. niger*, the effects of cDNA codon usage, signal peptide (original mnp2 versus *A. niger* Gla1), and the original cDNA with its first intron on *mnp2* on transgene expression were further examined [45–47]. Therefore, the *mnp2* transgene expression with its genomic DNA coding sequence (g*mnp2*, pZD4175), the *mnp2* gene with codon usage optimization for *A. niger* (co*mnp2*, pZD4172), the original mnp2 signal peptide replaced by the *A. niger* Gla1 signal peptide (Sgla1/co*mnp2*, pZD4173), or the original cDNA with its first intron (c*mnp2*/1st intron, pZD4176) was randomly integrated into *A. niger* 11414 *prtT*∆ strain. Furthermore, the transgene expression of *mnp3* (*gmnp3*, pZD4181), *mnp4* (*gmnp4*, pZD4174), and *mnp5* (*gmnp5*, pZD4198) with their original genomic coding regions were evaluated for their overall production under the control of the *A. niger gla1* promoter (*gla1*p) and the *A. niger gpdA* transcriptional terminator (*gpdA*t).

**Fig. 3:**
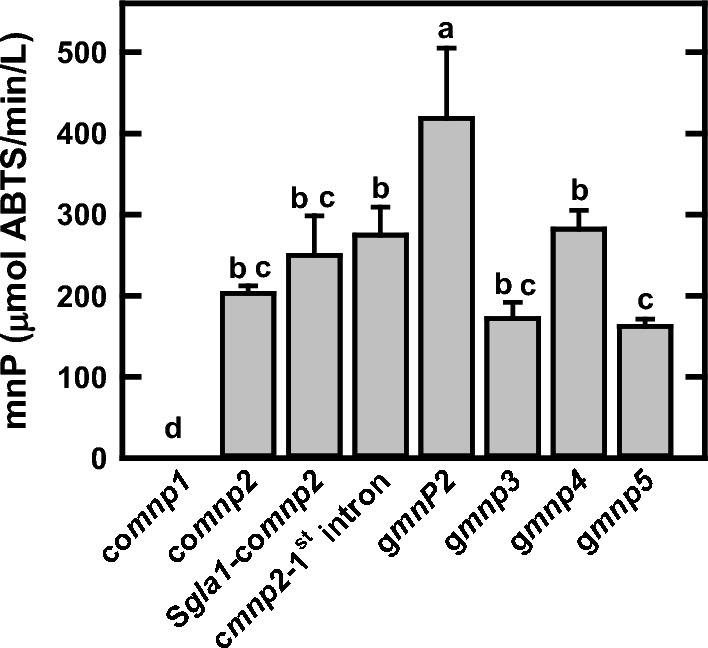
The manganese peroxidase (mnP) activities in the selected *A. niger* transgenic strains with the best transgene expression of the five *mnp* (*mnp1*, *mnp2*, *mmp3*, *mmp4*, and *mnp5*) genes from *P. chrysosporium*, *G. lucidum* gl*mnp1* gene, or *P. ostreatus* Po*mnp4* gene. *A. niger* codon usage optimization was applied to the cDNAs of *mnp1* (co*mnp1*), *mnp2* (co*mnp2*), Gl*mnp1* (coGl*mnp1*), and Po*mnp4* (coPo*mnp4*) genes. *A. niger* gla1 signal peptide was used to replace the original one of *mnp2* (Sgla1-co*mnp2*), Gl*mnp1* (Sgla1-coGl*mnp1*), or Po*mnp4* (Sgla12-coPo*mnp4*). The original cDNA with its first intron of *mnp2* was de novo synthesized (cmnp2-1st intron). The *gmnp2*, *gmnp3*, *gmnp4*, and *gmnp5* were prepared with their entire genomic coding sequences. The shaker flask cultures with modified minimal medium (mMM) and 2.5 g/L bHg were maintained at 30 °C and 200 rpm for 2 days. All data were averaged from three biological replicates. All data represent mean ± SD (*n* = 3 biological replicates). Different lowercase letters represent statistically significant differences (*p* < 0.05) using ANOVA and Tukey’s post hoc tests

The strain with the highest mnP activity for each transgene expression construct was selected for comparison. The results in Fig. 3 show that the transgene expression of g*mnp2* has the highest mnP activity (418 U/L). The transgene expression with the original cDNA and its 1st intron (275 U/L) is better than that with the cDNA codon optimization for *A. niger*. This indicates that the entire original *mnp2* gene with all introns can be effectively spliced by *A. niger* and achieve better transgene expression than that with the only first intron. The Gla1 signal peptide replacement of the original mnP2 one (Sgla1-comnP2) has 23.2% higher activity than that with its original peptide (comnP2), suggesting the Gla1 secretory signal peptide is more effective than mnp2 one for protein secretion, which was also observed in a previous study [48].

Additionally, the mnP activity is 172, 281, and 165 U/L for *mnp3*, *mnp4*, and *mnp5*, respectively, which are much lower than the activity for *mnp2*. No activity was detected for *mnp1* with ABTS as a substrate. However, the activity of mnp1 purified from *P. chrysosporium* could be quantified with 2,6-dimethoxyphenol (2,6-DMP) as a substrate [40, 49], suggesting that the mnp1 might have substrate specificity.

### Improvement of overall mnP production via reduction of protease degradation and protein secretory pathway amendment

Beyond the conditions examined above, the secretory pathway and extracellular stability are another two factors that affect ligninolytic enzyme production in *A. niger*. Several proteins involved in protein secretory pathway have been identified that impact the stability of the heterologous proteins in the filamentous fungal hosts [50, 51]. Their effects were mainly determined individually, while the synergistic study is now more feasible due to recent progress in the application of CRISPR–Cas9 based methods for *A. niger* [52]. One strategy for the improvement of heterologous protein production in *A. niger* is to reduce the secretion of extracellular proteases by *prtT* gene disruption [53, 54]. In addition, there is an array of proteins known to be involved in protein secretion processes [55]. For instance, yeast v-SNARE binding protein (*vsm1*) is a negative regulator of constitutive protein secretion [56].

**Fig. 4:**
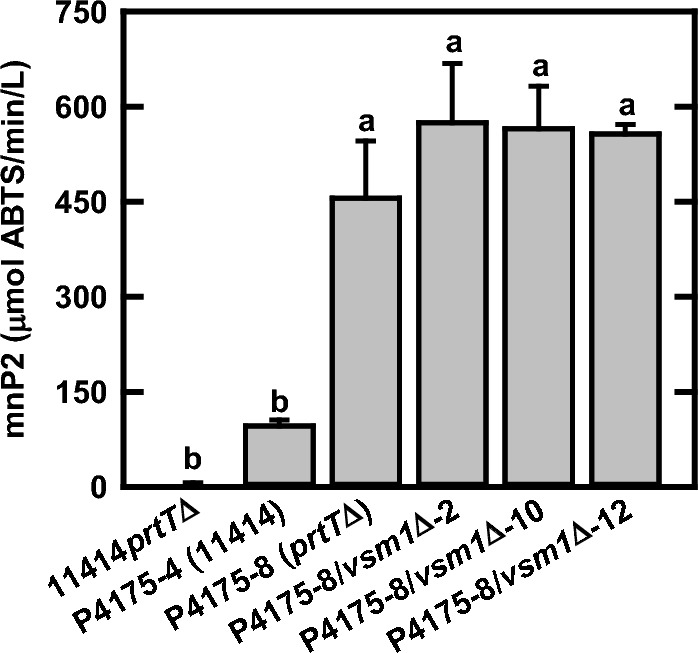
The effects of *prtT* and *vsm1* disruption on mnp2 production in *A. niger*. The entire genomic coding sequence of *mnp2* gene under the control of *gla1* promoter and *gpdA* transcriptional terminator was randomly integrated into the chromosomes of *A. niger* parent strain ATCC 11414 or 11414*prtT*∆ strain and resulted in the highest mnP production strain pZD4175-4 in *A. niger* ATCC 11414 and pZD4175-8 in the *prtT*∆ strain. The *vsm1* gene was further disrupted in the pZD4175-8 strain and resulted in pZD4175-8/*vsm1*D-2, -10 and -12 strains. The shaker flask cultures with modified minimal medium (mMM) and 2.5 g/L bHg were maintained at 30 °C and 200 rpm for 2 days. All data represent mean ± SD (*n* = 3 biological replicates). Different lowercase letters represent statistically significant differences (*p* < 0.05) using ANOVA and Tukey’s post hoc tests

In this study, we integrated the *mnp2* transgene expression cassette (pZD4175) into both the wild-type ATCC 11414 and the 11414 *prtT*∆ strain. The results in Fig. 4 show that disruption of *prtT* (pZD4175-8) led to approximately a fivefold improvement in mnP production in comparison with that of the transgenic expression in wild-type ATCC 11414 (pZD4175-4) strain. Further disruption of *vsm1* homolog gene in the pZD4175-8 transgenic strain led to another 30% increase in the mnP production. Our results demonstrate that reduction of both extracellular proteases (*prtT*D) and a potentially negative regulator (*vsm1*D) led to significant improvement of mnP production in *A. niger*.

### Transgene expression of laccase hybrid (Lac131) of *Trametes* sp. C30 in transgenic *A. niger*

Laccase (Lac), lignin peroxidase (LiP), manganese peroxidase (mnP), versatile peroxidase (VP), and dye-decolorizing peroxidase (DyP) function synergistically to depolymerize lignin in the lignocellulose biomass in nature [57]. For effective depolymerization of lignin in the lignocellulosic biomass, the lignin-degradation microorganisms always produce a set of different combinations of ligninolytic enzymes with multiple isozymes and isoforms that respond to a different environmental stimuli such as nutrient compositions, oxygen level, and growth temperature [58]. For example, 6 isoforms of LiP and 4 isoforms of mnP were purified from *P. chrysosporium* in nitrogen-limited culture conditions [59], and later the whole genome sequences confirmed that *P. chrysosporium* contains 10 *lip* genes and 5 *mnp* genes [39]. However, it is difficult to quantify the contribution of individual isoforms or isozymes to lignin depolymerization in native microbes due to their multiplex phenotypes. Therefore, combined transgenic expressions of selected ligninolytic enzymes in the single transgenic strain is one of the strategies to evaluate their roles and synergetic effects in the lignin depolymerization in an industrial, non-lignin-degrading microbe, such as *A. niger*.

**Fig. 5:**
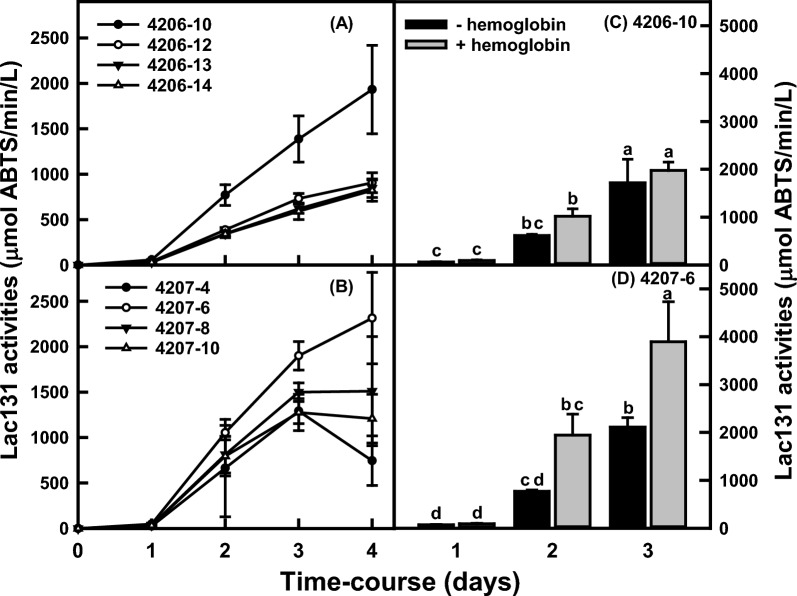
The time-course of Lac131 activities in the selected transgenic strains grown in the modified minimal medium (mMM) with or without 2.5 g/L bHg at 30 °C and 200 rpm. **A** The Lac131 activities of selected strains of pZD4206 series that were grown in mMM, where the *lac131* gene cDNA with *A. niger* codon usage optimization and under the control of *A. niger ubi1* promoter was randomly integrated into the chromosomes of pZD4175-8 strain. **C**, **D** Were the Lac131 activities of pZD4206-10 and pZD4207-6 strains that were grown in the mMM with or without 2.5 g/L bHg. All data represent mean ± SD (*n* = 3 biological replicates). Different lowercase letters represent statistically significant differences (*p* < 0.05) using ANOVA and Tukey’s post hoc tests (**C**, **D**)

The transgene expression of laccase hybrid (*lac131*) of *Trametes* sp. C30 was evaluated in both the transgenic pZD4175-8 (*mnp2*) strain and the *A. niger* 11414*prtT*∆ strain. The *lac131* with codon optimization for *A. niger* under the control of *ubi1* promoter and Gla1 signal peptide (co*lac131*/Sgla1; pZD4206) was integrated into the *mnp2* transgenic strain pZD4175-8 or under the control of *gla1* promoter (pZD4207) into the 11414*prtT*∆ strain. The laccase hybrid activity varies among the 14 transgenic strains for either pZD4206 or pZD4207 (data not shown). The time-course Lac131 activities were determined in four selected transgenic strains. Figure 5A, B shows that strain pZD4206-10 and pZD4207-6 have the highest laccase activity (1933 or 2315 U/L) among the selected transgenic strains, respectively. The effects of bHg on laccase production were also evaluated with the laccase production strains of pZD4206-10 & pZD4207-6 (Fig. 5C, D). After 2 days of growth, the laccase activity was about 66% higher in the transgenic strain pZD4206-10 grown with 2.5 g/L bHg (1022 U/L) than that without bHg (617 U/L), but the difference was not significant after 3 days of growth. In contrast, the laccase activity was 153% and 84% higher in the transgenic strain pZD4207-6 in the culture with bHg (1945 & 3893 U/L) than without bHg (770 & 2116 U/L) after 2 or 3 days growth, respectively. The results show that *Trametes* sp. C30 *lac131* transgene expression was much higher than the mnP in *A. niger* and bHg also exhibits its positive effects on lac131 production.

### Evaluation of mnP and laccase hybrid decolorization of selected aromatic dyes

**Fig. 6:**
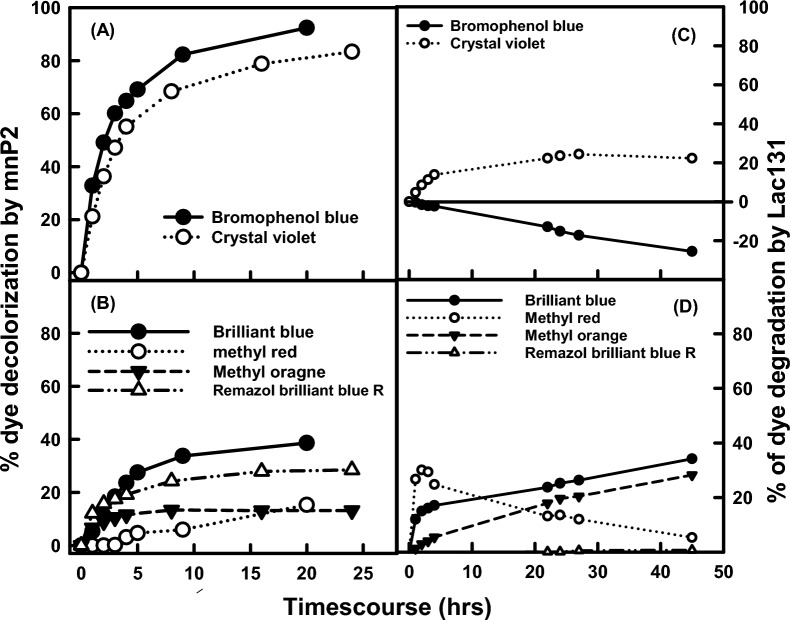
The time-course of decolorization of selected aromatic dyes by mnP or Lac131 culture supernatants that pZD4175-8 or pZD4207 strain was grown in the modified minimal medium (mMM) with 2.5 g/L bHg at 30 °C and 200 rpm. The bromophenol blue and crystal violet (**A**) or brilliant blue, methyl red, methyl orange, or Remazol brilliant blue R (**B**) were decolored by mnP; while bromophenol blue and crystal violet (**C**) and the brilliant blue, methyl red, methyl orange, or Remazol brilliant blue R (**D**) were decolored by laccase hybrid. All data were averaged from five technical replicates

While the mnP and laccase are known for their roles in lignin depolymerization, it is a great challenge to quantify their delignification activity. One way to evaluate their activities is by monitoring the changes in aromatic dyes [60, 61]. The mnP oxidizes manganese (Mn^2+^) to Mn^3+^ which acts as a diffusible oxidant to break down lignin and various organic compounds, while laccase takes one electron from the hydroxyl group of the phenolic compounds and followed by a deprotonation reaction. In this study, the aromatic dyes brilliant blue, bromophenol blue, crystal violet, methyl orange, methyl red, and Remazol brilliant blue R were selected to examine the effectiveness of the mnP and laccase hybrid for dye decolorization. The culture supernatants from strains of pZD4175-8 (~ 12.5 U mnP) and pZD4207-6 (~ 100 U Lac131) were used for decoloring the selected dyes. The results in Fig. 6A show that mnP effectively decolorizes the bromophenol blue and crystal violet up to 60 and 47% in the first 3-h treatment and reached 92 and 80% after 20-h incubation, respectively. It also decolorized brilliant blue, methyl Remazol brilliant blue R, methyl orange, or methyl red in various degrees (38.6, 28.5, 24.0, or 15.3%, respectively) after 20–24 h. In contrast, the laccase hybrid in Fig. 6C exhibits low activity in decoloring crystal violet (34.2%) and no effects on bromophenol blue or intense dye color, which may arise from deprotonation by the laccase reaction. The laccase hybrid could decolorize methyl orange (28.3%) and brilliant blue (22.3%) (Fig. 6D). The methyl red was quickly decolorized by laccase hybrid to 30% in 2 h, while recovered back to only 5.3% after 45-h incubation (Fig. 6D), suggesting both protonation and deprotonation without significant structure changes. However, the laccase hybrid could not decolorize the Remazol brilliant blue R. This demonstrates that mnP is much more effective than the laccase hybrid to decolorize those selected aromatic dyes by their distinct processes.

### Activity of mnP and laccase hybrid with NIMS-tagged lignin dimers

**Fig. 7:**
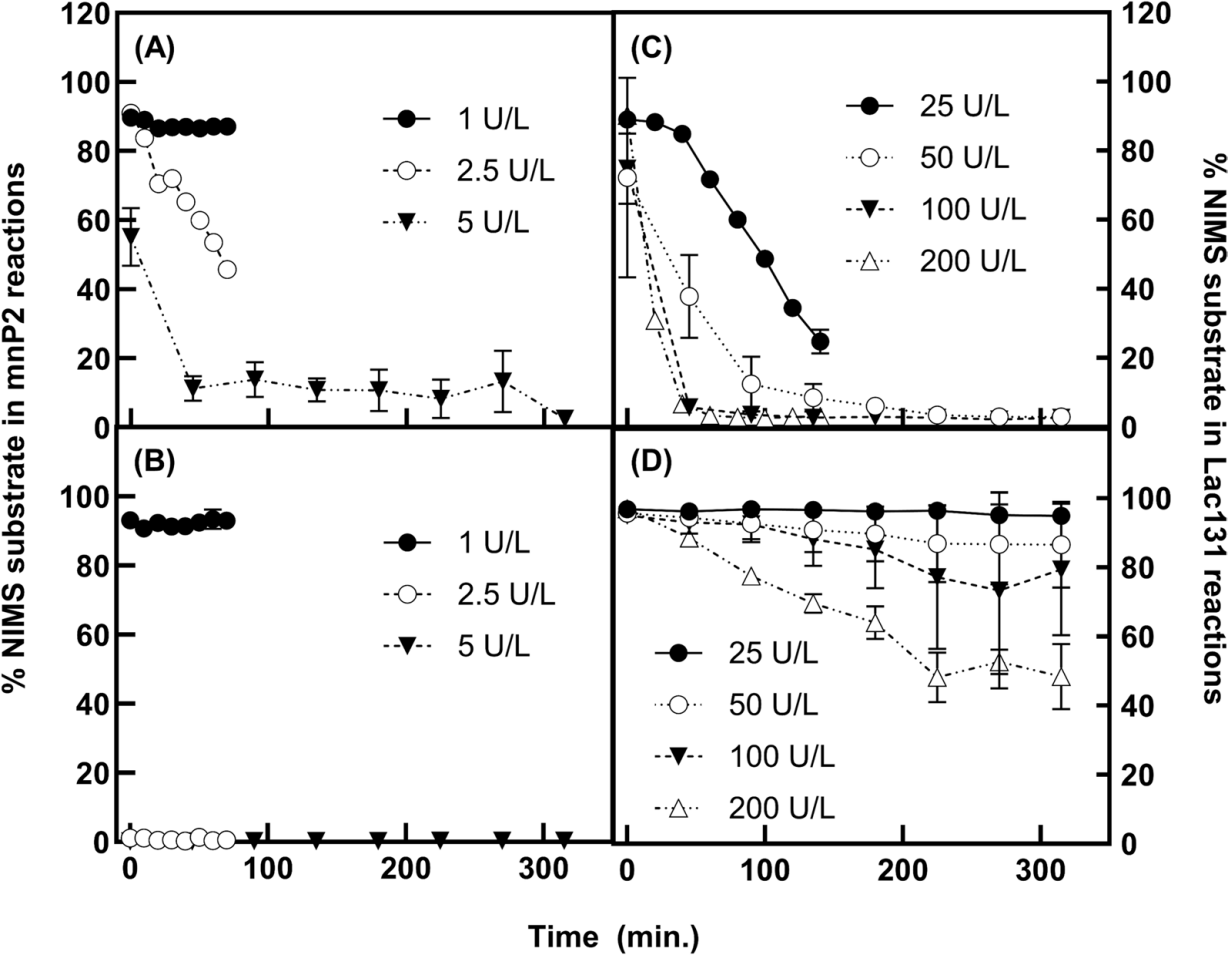
The time-courses of NIMS-tagged model lignin dimer cleavage by mnP2 or Lac131 culture supernatants. NIMS-tagged dimers representing the beta-O-4’ bond (**A**, **C**) and the beta-beta’ bond (**B**, **D**) were tested. Both mnP2 (**A**, **B**) and Lac131 (**C**, **D**) reduced the relative percentage of both substrates. Relative percentage is shown on both y-axes and was calculated by dividing the peak intensity of the substrate peak by the sum of peak intensities for the substrate and product peaks. Specific products formed during the reactions are shown in Supplementary Figs. 3 and 4

To determine the ability of mnP and the laccase hybrid to break the bonds found in lignin, both enzymes were tested with NIMS-tagged model lignin dimers representing the beta-O-4’ and beta-beta’ bonds in lignin. The NIMS tag contains a polyfluorinated moiety that enables rapid sample cleanup and ion detection on the NIMS chip and a cation moiety that improves ionization. The NIMS tag reduces polymerization reactions and allows bond-specific analysis of cleavage activity [62]. Both the laccase and mnP cleaved the beta-O-4’ and beta-beta’ substrates with the oxidation-cleavage pathways published previously [Fig. S4 & S5; [35]]. The mnP cleaved approximately 90% of the beta-O-4’ substrate rapidly at relatively low enzyme concentrations (5 U/L) within an hour (Fig. 7A), whereas 50 U/L laccase was required to cleave the substrate this timeframe (Fig. 7C). The laccase was much slower to cleave the beta-beta’ substrate than the beta-O-4’ substrate, requiring over three hours to cleave approximately 50% of the substrate at an enzyme concentration of 200 U/L (Fig. 7D). Interestingly, 2.5 U/L mnP cleaved the beta-beta’ substrate much more rapidly than the beta-O-4’ substrate, with 100% of the substrate depleted before 10 min (Fig. 7B). This may suggest that the mnP is more effective than the laccase to deconstruct the lignin complex.

## Conclusions

In summary, by ABTS analysis, four out of five manganese peroxidase (*mnp*) genes from *P. chrysosporium*, and *Trametes* sp. C30 laccase hybrid (*lac131*) were functionally expressed in *A. niger* under the control of strong *gla1* promoter. Transgene expression of the intact g*mnp2* genomic coding region led to the highest mnP production in *A. niger* as compared to other genetic engineering strategies, such as codon usage optimization, the original cDNA of *mnp2* with its first intron, or signal peptide. Disruption of both *prtT* and the *vsm1* homolog genes led to a sixfold improvement in mnP activity in the g*mnp2* transgenic *A. niger* strain. In contrast to prior work, we observed that hemin had no effects on mnP production in *A. niger*. Supplementing bHg, skimPs, soyPs or BSA into the cultures substantially increased mnP production to different degrees. The optimal shaker speed, culture temperature and initial medium pH were determined as 200 rpm, 25 °C and pH4.5. Further optimization of the culture conditions is necessary to reduce the requirement for extra protein supplements. Both mnP and laccase hybrid Lac131 decolorized selected aromatic dyes and cleave NIMS-tagged model lignin dimers, but mnP was more effective than the laccase hybrid. Co-expression of variety of ligninolytic enzymes in the same *A. niger* transgenic strain will enhance lignin bioconversion to value-added chemicals, such as malic acid or 3-hydroxypriopionic acid.

## Supplementary Information

Supplementary material 1: Table S1: Oligos used for transgene vector constructions of the gene over-expressions or disruptions. Figure S1: The diagram of twelve different transgene expression constructs were prepared with oligo pairs listed in Table S1. 1. The diagram of Phanerochaete chrysosporium mmp1 gene coding sequence without original protein secretory signal peptide (orSP) with codon usage optimization for A. niger under the control of A. niger gpdA promoter, A. nidulans trpC transcriptional terminator, A. niger gla1 secretory signal peptide (Sgla1) and E. coli hygromycin B phosphotransferase (hph) selection marker with gla1 locus targeting; 2. The diagram for prtT disruption construct with bleomycin resistance (ble) gene selection marker; 3. The diagram of P. chrysosporium mmp2 gene transgene expression construct with the A. niger codon usage optimization and orSP; 4. The diagram of P. chrysosporium mmp2 gene transgene expression construct with A. niger codon usage optimization, Sgla1, and hph selection marker; 5. The diagram of P. chrysosporium mmp2 gene transgene expression construct with the orSP, original cDNA and the first original intron and hph selection marker; 6. The diagram of P. chrysosporium mmp4 gene transgene expression construct with the orSP and the original genomic DNA coding region and hph selection marker; 7. The diagram of P. chrysosporium mmp2 gene transgene expression construct with the orSP and the original genomic DNA coding region and hph selection marker; 8. The diagram of P. chrysosporium mmp2 gene transgene expression construct with the orSP and the original genomic DNA coding region fused with gfp and hph selection marker; 9. The diagram of P. chrysosporium mmp3 gene transgene expression construct with the orSP and the original genomic DNA coding region and hph selection marker; 10. The diagram of P. chrysosporium mmp5 gene transgene expression construct with the orSP and the original genomic DNA coding region and hph selection marker; 11. The diagram of Trametes sp. C30 laccase hybrid (lac131) gene transgene expression construct with Sgla1 and the A. niger codon usage optimization under the control of A. niger ubi1 promoter and nourseothricin (nat1) selection marker; 12 The diagram of Trametes sp. C30 laccase hybrid (lac131) gene transgene expression construct with Sgla1 and the A. niger codon usage optimization under the control of A. niger gla1 promoter and nat1 selection marker. Figure S2: The Ethidium bromide agarose gel shows the three selected prtTD transgenic strains confirmed by PCR with oligo pair 576BleF/2522ScrR and the strain prtTD-3 was used as 11414prtTD strain. The lane-1 is the BstII lDNA marker. Lane-2, 3, & 5 are selected prtTD strains, while lane-4 is the parent stain ATCC 11414. Figure S3: The GFP observation in the mnp2-gfp fusion expression. The upper panel is the parent 11414prtT∆ strain and the lower panel is the selected transgenic strain pZD4180-1. Figure S4. The time-courses of NIMS-tagged model lignin dimer representing the beta-O-4’ bond. Scheme of NIMS-tagged beta-O-4’ oxidation and cleavage adapted from Deng et al. 2018 (A). Beta-O-4’ substrate conversion by 1, 2.5, 2.5, and 5 U/L mnP2 (B-E) and 25, 50, 100, and 200 U/L Lac131 (F-I). Relative proportions are shown on y-axes and were calculated by dividing the peak intensities by the sum of peak intensities for the substrate and product peaks. Figure S5. The time courses of NIMS-tagged model lignin dimer representing the beta-beta’ bond. Scheme of NIMS-tagged beta-beta’ oxidation and cleavage adapted from Onley et al. 2025 [35] (A). Beta-beta’ substrate conversion by 1, 2.5, and 5 U/L mnP2 (B-D) and 25, 50, 100, and 200 U/L Lac131 (E-G). Relative proportions are shown on y-axes and were calculated by dividing the peak intensities by the sum of peak intensities for the substrate and product peaks.

## Data Availability

No datasets were generated or analyzed during the current study.

## Abbreviations

bHg: Bovine hemoglobin
BSA: Bovine serum albumin
CM: *Aspergillus niger* Complete medium
Lac131: The laccase hybrid from lac131 transgene expression
mMM: Modified *A. niger* minimal medium
mnP: Manganese peroxidase
mnP2: The mnP protein from *mnp2* transgene expression
skimPs: Skim milk proteins
soyP: Soy proteins
