# Supplementary material for: Optimal production of Phanerochaete chrysosporium manganese peroxidases and Trametes sp. C30 laccase hybrid Lac131 in Aspergillus niger for lignin bioconversion
Source: Biotechnol Biofuels Bioprod. 2025 Oct 6;18:102. doi: 10.1186/s13068-025-02690-x (PMC12502335; doi:10.1186/s13068-025-02690-x)

**Supplementary Table 1.** Oligos were used for transgene expression constructs

| Oligo name | Transgene expression construct |
| --- | --- |
|  | prtTΔ (2) |
| 2515prtF1 | ggtcgacggtatcgatagagctcGGAAGCAGCGGGATTTAGTTAC |
| 2516prtR1 | tgcacctcagTCCATCGAGGCGGTTTCATC |
| 2517bleF | cctcgatggaCTGAGGTGCAGTGGATGATTATTAATC |
| 2518bleR | actcagcagcTTGATCTGCTTGATCTCGTC |
| 2519prtF2 | agcagatcaaGCTGCTGAGTGGTATCTCAATTAAC |
| 2520prtR2 | agtggatcccccgggctgcaTTTCCAACCCGATCTCGATTAC |
| 2521scrf1 | gcggcagttcctcactaatttc |
| Ble-293 | cgtcggtcagtcctgctcctc |
| Ble-292 | caagttgaccagtgccgttcc |
| 2522scrr1 | cctttcgttgcacgtcatcat |
|  |  |
|  | pZD4172/pZD4173 (3/4) |
| 2883glaF | cgaggtcgacggtatcgataGAGGAAATCTCCCCTGATCTTC |
| 2893PglaR | caaaggccatTGCTGAGGTGTAATGATGCTG |
| 2894ocmnp2F | cacctcagcaATGGCCTTTGGGTCGCTACTAG |
| 2895ocmnp2R | cctagtttcaCGCTGGACCATCGAATTGAAC |
| 2896gpdAtF | tggtccagcgTGAAACTAGGAATCAGGACGG |
| 2886ampR | CAATAGGTCAGGCTCTCGCTG |
| 2887hphF | AGCGAGAGCCTGACCTATTG |
| 2888hphR | agtggatcccccgggctgcaGGACTGTCTGTCTGGTCTTC |
| 2897glaR | gacacacagcCTTGGAAATCACATTTGCCAAC |
| 2898ocmmnp2bF | gatttccaagGCTGTGTGTCCGGATGGAAC |
|  |  |
|  | pZD4174/pZD4175/pZD4176/pZD4180 (5/6/7/8) |
| 2931PglaR | cgaaggccatTGCTGAGGTGTAATGATGCTG |
| 2932gmnP2F | cacctcagcaATGGCCTTCGGTTCTCTCCTC |
| 2933gmnP2R | cctagtttcaCGACACTGAAGGCGTAATACTG |
| 2934gpdAtF | ttcagtgtcgTGAAACTAGGAATCAGGACGG |
| 2909mnp2R | cctagtttcaAGGTGCATTGCCGGAGGTGAG |
| 2910gpdAtF | caatgcacctTGAAACTAGGAATCAGGACGG |
| 2966mnP2R2 | agaggttctcGGCAGGGCCATCGAACTGAAC |
| 2967GFPF | tggccctgccGAGAACCTCTATTTCCAGGG |
| 2968GFPR | AGGGCGAACTTAAGAAGGTATG |
| 2969hphF2 | CATACCTTCTTAAGTTCGCC |
|  |  |
|  | pZD4181/pZD4198 (9/10) |
| 2883glaF |  |
| 2954glaR | gaaacgccatTGCTGAGGTGTAATGATGCTG |
| 2955mnp3F | cacctcagcaATGGCGTTTCCATCCTCACTC |
| 2956mnp3R | cctagtttcaTCAGACTTATGCAGGGCCGTTG |
| 2957hphF | ataagtctgaTGAAACTAGGAATCAGGACGG |
| 2958glaR | aaaacgccatTGCTGAGGTGTAATGATGCTG |
| 2959mnp5F | cacctcagcaATGGCGTTTTCCACCCTATTG |
| 2960mnp5R | cctagtttcaTTAGGCCGGACCATCAAACTG |
| 2961hphF | tccggcctaaTGAAACTAGGAATCAGGACGG |
|  |  |
|  | pZD4206/pZD4207 (11/12) |
| 2680nat1F | aggtcgaggtcgacggtatcgataACAGAAGATGATATTGAAGGAGC |
| 3061nat1-ubi1pR | ggaacgacatCTTGATGAAGGTCTGGGTTG |
| 3062Lac131F | cttcatcaagATGTCGTTCCGATCTCTTCTCGC |
| 3063Lac131R | gccgaagacctcaTTGGTCGTCCGGGCTGAGTG |
| 3064actrpCtF | ggacgaccaatgaGGTCTTCGGCTATAGTTCATTTTTATC |
| 2675actrpCtR | agtggatcccccgggctgcaGTTGCGATCAGGTGTGTAATTG |
| 3065glaF | cgaggtcgacggtatcgataATCCGAACTCCAACCGGGGG40 |
| 3066lac131R | gccgaagaccTCATTGGTCGTCCGGGCTGAG |
| 3067actrpcF | cgaccaatgaGGTCTTCGGCTATAGTTCATTTTTATC |
| 3068actrpcR | catcttctgtGTTGCGATCAGGTGTGTAATTG |
| 3069nat1F | tgatcgcaacACAGAAGATGATATTGAAGGAGC |
| 3070nat1R | agtggatcccccgggctgcaCAGTAAGTAGAAAGCTTTGGG |
|  |  |
|  | CRISPR-Cas9 for pGY35 |
| Cas9-vsm1-1 | TAGGTGTTCAGGTTACCCTGCGG |
| Cas9-vsm1-2 | GTTGTCGTTGAAGGACATCACGG |
|  |  |
| 2675TrpCR | agtggatcccccgggctgcaGTTGCGATCAGGTGTGTAATTG |

**Supplementary Figure 1.** Twelve different transgene expression constructs were prepared with oligo pairs listed in the supplementary table (ble: bacterial bleomycin resistance gene; Co: codon usage optimization for *Aspergillus niger*; cI: cDNA with the first intron of *Phanerochaete chrysosporium mnp2* gene; g: genomic; *gfp*: green fluorescence protein gene; *gpdA*p: *Aspergillus niger* *gpdA* gene promoter; *gpdA*t: *A. niger* *gpdA* gene transcriptional terminator; *hph*: bacterial hygromycin B phosphotransferase gene; mnP: manganese peroxidase; orSP: original signal peptide of mnP proteins from *P. chrysosporium*; *prtT*: transcriptional activator of proteases prtT gene; Sgla1: Gla1 signal peptide from *A. niger*; *trpC*t: *Aspergillus nidulans trpC* transcriptional terminator).


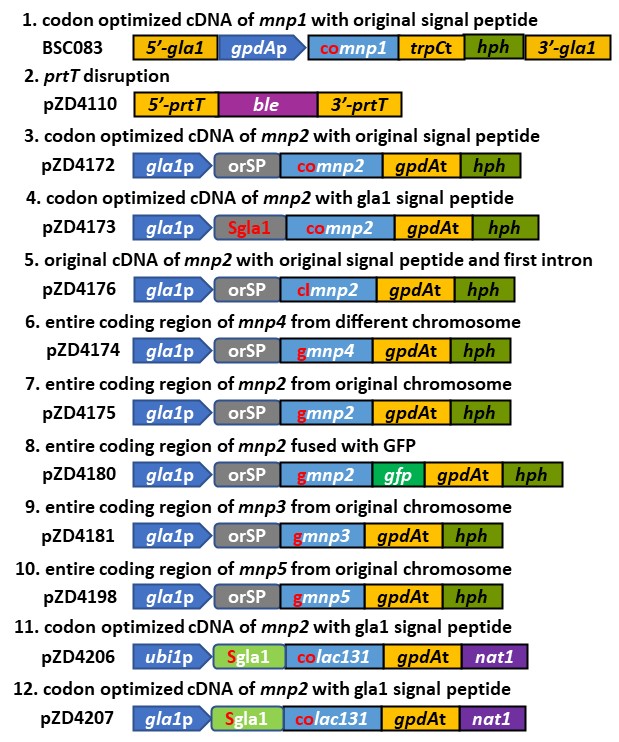


**Supplementary Figure 2.** Four selected *prtT*Δ transgenic strains confirmed by PCR with oligo pair 2521/. Strain-4 was used as 11414*prtT*Δ strain.


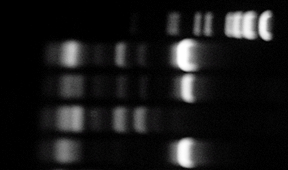


**BstII λDNA marker**

**Strain-1**

**Strain-2**

**Strain-3**

**Strain-4**

**Supplementary Figure 3.**  The GFP observation in the *mnp2-gfp* fusion expression


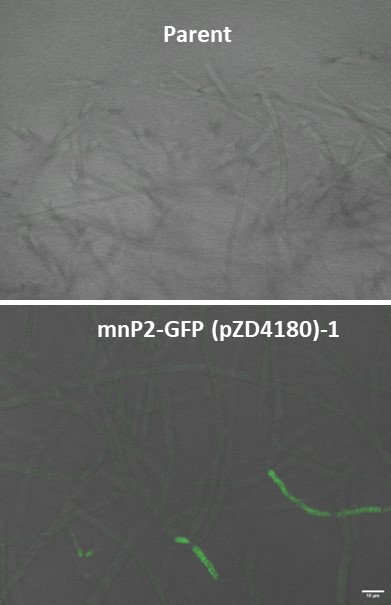


**Supplementary Figure 4.** Detailed NIMS analysis-1


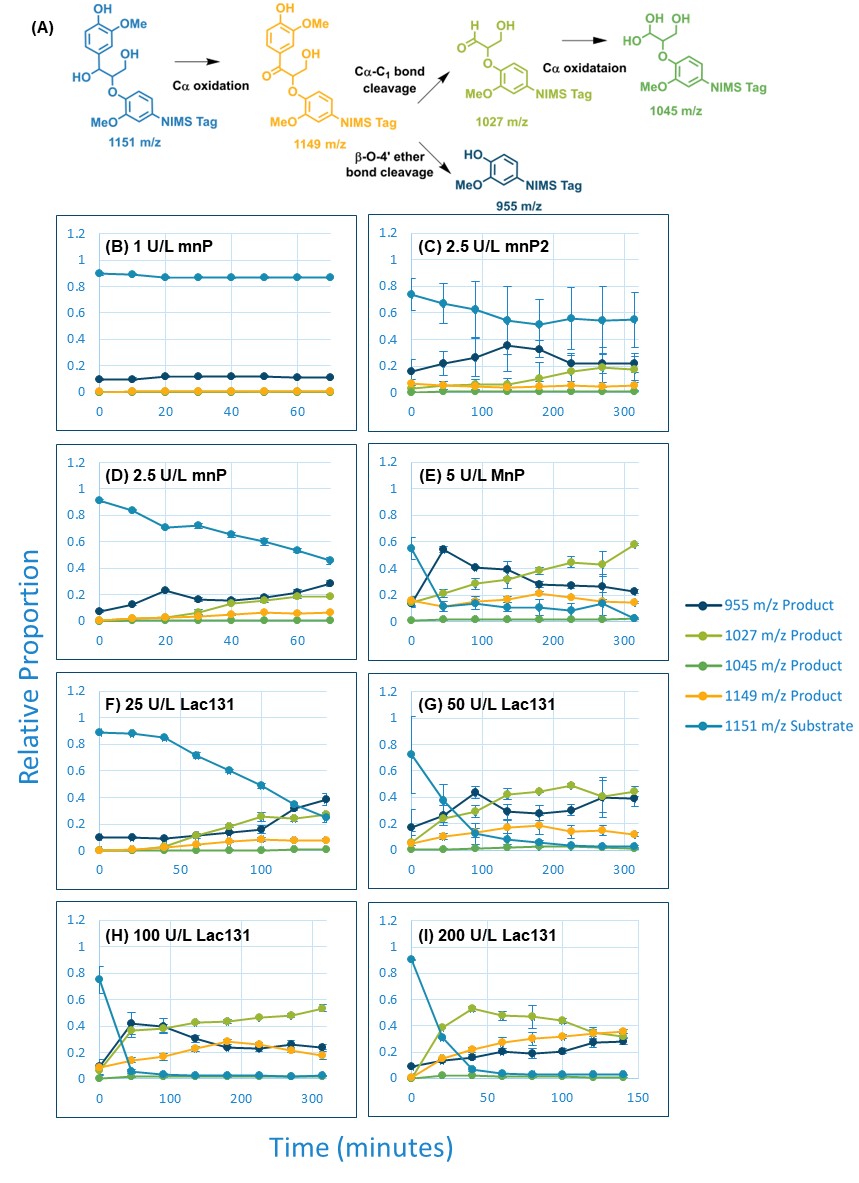


**Supplementary Figure 5.** Detailed NIMS analysis-2


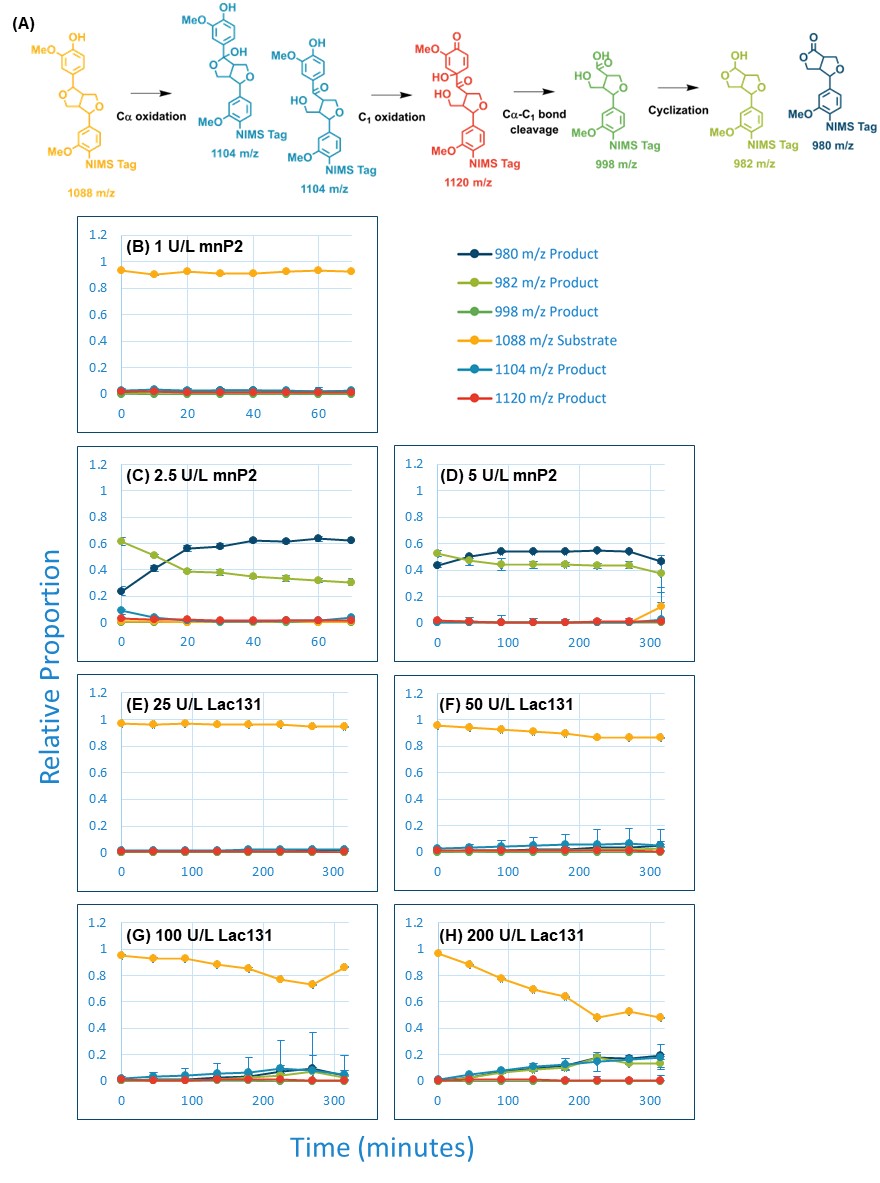

Supplement: Supplementary file 1 — Supplementary material 1: Table S1: Oligos used for transgene vector constructions of the gene over-expressions or disruptions. Figure S1: The diagram of twelve different transgene expression constructs were prepared with oligo pairs listed in Table S1. 1. The diagram of Phanerochaete chrysosporium mmp1 gene coding sequence without original protein secretory signal peptide (orSP) with codon usage optimization for A. niger under the control of A. niger gpdA promoter, A. nidulans trpC transcriptional terminator, A. niger gla1 secretory signal peptide (Sgla1) and E. coli hygromycin B phosphotransferase (hph) selection marker with gla1 locus targeting; 2. The diagram for prtT disruption construct with bleomycin resistance (ble) gene selection marker; 3. The diagram of P. chrysosporium mmp2 gene transgene expression construct with the A. niger codon usage optimization and orSP; 4. The diagram of P. chrysosporium mmp2 gene transgene expression construct with A. niger codon usage optimization, Sgla1, and hph selection marker; 5. The diagram of P. chrysosporium mmp2 gene transgene expression construct with the orSP, original cDNA and the first original intron and hph selection marker; 6. The diagram of P. chrysosporium mmp4 gene transgene expression construct with the orSP and the original genomic DNA coding region and hph selection marker; 7. The diagram of P. chrysosporium mmp2 gene transgene expression construct with the orSP and the original genomic DNA coding region and hph selection marker; 8. The diagram of P. chrysosporium mmp2 gene transgene expression construct with the orSP and the original genomic DNA coding region fused with gfp and hph selection marker; 9. The diagram of P. chrysosporium mmp3 gene transgene expression construct with the orSP and the original genomic DNA coding region and hph selection marker; 10. The diagram of P. chrysosporium mmp5 gene transgene expression construct with the orSP and the original genomic DNA coding region and hph selection m [file 13068_2025_2690_MOESM1_ESM.docx]
